# Practical tips for bedside teaching of infectious diseases practice to junior doctors

**DOI:** 10.15694/mep.2017.000180

**Published:** 2017-10-11

**Authors:** Sharavan Sadasiv Mucheli, Sophia Archuleta

**Affiliations:** 1 Tan Tock Seng Hospital; 2 National University Hospital

**Keywords:** Junior Doctors, Infectious Diseases, Bedside teaching, Clinical teaching microskills

## Abstract

This article was migrated. The article was marked as recommended.

Infectious diseases, being a cognitive specialty, is commonly perceived to be difficult to teach at the bedside. Most junior doctors have had variable amounts of exposure to infectious diseases as medical students, primarily in the form of lectures as part of a microbiology curriculum. Teaching principles of infectious diseases practice to junior doctors is essential to develop good antibiotic prescribing skills. We suggest a practical guide with 3 x 3 steps to teach infectious disease practice at the bedside. We have attempted to outline the anticipated problems with the instruction of Infectious disease practice in the clinical setting and then have proceeded to adopt an expanded version of the clinical micro-skills model to address those gaps.

## Introduction

Infections are probably the most frequent issue that affects hospitalized patients ( Nicolle et al., 1999), hence work-place based teaching of infectious disease (ID) practice to junior doctors is an important task. Teaching principles of infectious diseases practice to junior doctors is quintessential to develop good antibiotic prescribing skills ( Pulcini & Gyssens, 2013). It could have a tangible impact on the future of clinical practice and hence the time invested in it would be worthwhile.

Infectious diseases, being a cognitive specialty, is commonly perceived to be difficult to teach on the wards. It often requires wide-base of knowledge and an expertise in synthesizing and analyzing varied amounts of information to make a clinical decision. And most junior doctors have had variable amounts of exposure to Infectious diseases practice as medical students depending on the curriculum design.

## Barriers for teaching ID at the bedside

The potential pitfalls or barriers for effective bedside teaching of infectious diseases are listed below:

• Cognitive overload - Emphasis on too much knowledge can result in learner being overwhelmed/distracted
• Complexity in the form of diagnostic uncertainty inherent to Infectious disease practice requires sound clinical reasoning skills
• Disinterest may arise as the learner may perceive it to be not relevant, hence the need to make ‘relevance’ visible to appreciate and internalize topics

## Clinical teaching microskills

The five-step ‘microskills’ model ( Neher, Gordon, Meyer, & Stevens, 1992) has been an essential tool for most clinical teachers in bedside teaching. We propose an expanded version of the five-step micro-skills to facilitate ID teaching at the bedside. This is targeted at both ID specialists and non-ID specialists who manage common community-acquired and hospital-acquired infections. It is crucial to understand that the selection of cases should pertain to practice, which adheres to the pre-defined learning objectives. These skills can be used in cases with both differentiated and undifferentiated infectious problems.

For effective clinical teaching, it is important for the preceptor/clinical teacher to quickly assess the learner, provide feedback for improvement and take the opportunity to educate learner or teach important points (which is depicted in the table in 3 parts).

## Outline of steps for clinical teaching

**Table:** 

PART	RATIONALE	REMARKS
A	Assess learner, especially his/her knowledge and clinical reasoning skills	Learner-driven, guided by clinical teacher as necessary
B	Bridge the knowledge gaps, while minimizing cognitive load and helping transit over to clinical practice	Learner or Teacher-driven, depending on ‘learner diagnosis’. This step is of contextual relevance to teaching ID at the bedside.
C	Consolidate learning points and provide constructive feedback	Teacher-driven with learner participation

## Part A - Assess learner

### Step 1. What is the source of this infection?

*Alternatively, What is the infectious syndrome? (Get commitment)*

This is perhaps the most crucial question of part A. It is all the more relevant to Infectious diseases practice as often, junior doctors tend to react to ‘fever’ reflexively without exploring the likely cause behind and committing to a diagnosis. Commitment aids with learner diagnosis and helps pitch the teaching to the right level. Hesitancy in the learner to commit may also indicate lower confidence levels. A wrong diagnosis might expose lack of knowledge or faulty reasoning skills.

*E.g.*

*Case 1 - This patient has a ‘Urinary tract infection’*

*Case 2 - This patient has an ‘acute febrile illness with rash and conjunctivitis’*

### Step 2. Why do you say so? (Asking for justification)

Asking for justification explores clinical reasoning and helps do a case formulation. This question further elicits the illness scripts of the learner and their ability to apply it in a clinical setting. Inability to provide a sound justification for providing more insight into the learner’s capability. This step may further be used to help the learner give ‘problem representation’ with semantic qualifiers ( Cox, Irby, & Bowen, 2006), another useful way to stimulate clinical reasoning.

*E.g.*

*Case 1 - This middle-aged lady with poorly-controlled diabetes has 3-day history of fever with chills, lower urinary tract symptoms of dysuria and frequency, clinically demonstrable renal punch with pyuria on urine dipstick (A case of UTI/Pyelonephritis)*

*Case 2 - This young man, who is a migrant worker with unknown vaccination history has a 2-day history of fever with conjunctivitis, coryza and a morbilliform rash that spread in a centrifugal fashion (Measles)*

### Step 3. What are the other possibilities and why did you consider them? (Explore alternatives/Differentials)

This step of part A asks the learner to provide a list of differentials with a brief justification. It is much more relevant/pertinent in ‘undifferentiated’ cases. We feel that this is crucial as it avoids the pitfall of certain cognitive biases such as premature closure, attribution bias, and representation bias. This step also helps the learner appreciate that uncertainty or ambiguity is part of clinical practice and helps deal with it. A brief argument or reasoning exercise with ‘why and why not’ for alternatives also sharpens the learner’s cognitive skills.

*E.g.:*

*Case 1 - Another possibility to explain her symptoms is pelvic inflammatory disease with urethritis (as she has a backache with fevers, besides dysuria)*

*Case 2 - Possible differentials for this young man with fever, rash, conjunctivitis include an adenoviral infection (given the history of sick contact with a child), ZIKAvirus infection (given the demographics and general condition) etc.*

## Part B - Bridge knowledge gaps

### Step 4. What are the suspect culprit pathogens? (Linking microbiology at bedside)

We often find learners lacking in knowledge in this aspect. This step helps bridge knowledge gaps in the learner, and as it is ‘situational’, the learner is more likely to make a meaningful connection. This step also provides the learner with a clear, systematic approach to management of infections. It is important for the teacher to remember that the idea is not to provide an exhaustive list of organisms and overwhelm the learner (avoid cognitive loading). Instead, a prioritized list of ‘pathogens’ makes for better teaching.

*E.g.:*

*Case 1 - Uropathogens such as E.coli, Klebsiella, Proteus, and Enterococci are likely (UTI).*

*Case 2 - Viruses that cause exanthematous fevers such as Measles, Rubella, Parvovirus B19.*

### Step 5. What are the possible complications of this infection? (Anticipatory thinking)

When it comes to managing infections, just a cross-sectional view is often not adequate. As the progress with most infections is dynamic, it is important for the learners to anticipate complications. This also tends to be the less pondered upon aspect of infectious disease management. Anticipatory thinking helps prepare for likely untoward consequences and identify them early when they do occur ( Islam, Weir, Jones, Del Fiol, & Samore, 2015).

E.g.:

*Case 1 - If fever remains persistent in this lady with pyelonephritis, complications such as renal parenchymal abscesses, perinephric collections, urinary obstruction etc,.need to be considered.*

*Case 2 - Measles may result in severe complications including pneumonia and encephalitis.*

### Step 6. How would you manage this patient? (Principles of management)

In response to this question, most often, junior doctors would mention about the choice of antibiotics. This is an opportunity to introduce evidence-based principles for management of infections. Making ‘visible’ the rationale of antibiotic choice will inculcate good antibiotic stewardship principles, especially since this will be based on local epidemiology. Besides use of antibiotics for infection management, source control/evaluation when appropriate (such as surgical debridement, drainage of abscesses etc,.) needs to be emphasized.

*E.g.:*

*Case 1 - In our setting, given the high/low prevalence of ESBL pathogens (quote percent, when possible) as the cause for community-onset UTIs, we prescribe so-and-so antibiotics. It is important to address the predisposing factors, as in this lady’s case, diabetic control, and good perineal hygiene so as to prevent future episodes of pyelonephritis.*

*Case 2 - Management of Measles is mostly supportive. As there are concerns for hospital transmission, infection control measures need to be instituted or the relevant authorities need to be notified immediately.*

## Part C - Consolidate and give constructive Feedback

### Step 7. Take the opportunity to teach one/some general rules (Teaching point)

We like to limit our teaching points to 2 or 3 per case. We recommend that clinical teachers resist the urge to make this a lecture. This is not meant to be a discourse on an infectious syndrome or disease condition. The previous steps would have covered the relevant points related to patient’s diagnosis already.

*E.g.: Catheter associated-UTI is often a diagnosis of exclusion, as pyuria and bacteriuria need not signify infection. Changing a urinary catheter is an important aspect of treating a CA-UTI, as it removes the potential source of infection.*

### Step 8. Reinforce what was good (Positive reinforcement)

Acknowledge and reinforce good points observed during the interaction - be it presentation skills, clinical reasoning or a sound management plan. It will help with the confidence of the learner and create a positive learning environment ( Ramani & Krackov, 2012). Reinforcing learner’s actions when not deliberate might help establish them firmly. We recommend that the feedback provided be succinct and formative.

### Step 9. Correct mistakes (Feedback) and decide on an action plan

Unattended mistakes are likely to perpetuate. It is ideal if the mistakes are identified by learner itself. In such case, they are more likely to be receptive for advice. When learners are not mindful of mistakes made, it is important to cite a specific example, and correct in a neutral tone while being respectful to the learner. End by deciding on an action plan and provide resources for further reading when possible.

## Conclusion

Although this ‘expanded’ skills model requires more time (typically it requires about 6-8minutes per case) as compared to the one-minute preceptor, we believe it teaches infectious disease practice to junior doctors in a succinct yet comprehensive manner at the bedside, by addressing some of the common pitfalls.

## Notes On Contributors

***Dr. Sharavan Sadasiv Mucheli*** is a senior resident in the National Healthcare group Infectious Diseases program. He is an aspiring medical educator and is pursuing his Masters in Medical education with University of Dundee via distance learning. He is also a clinical tutor at the Lee Kong Chian School of Medicine and a clinical lecturer with Yong Loo Lin School of Medicine of the National University of Singapore. He is interested in bedside teaching and teaching clinical reasoning skills to learners at the bedside.

***Dr. Sophia Archuleta*** is an assistant professor in the Department of Medicine, Yong Loo Lin School of Medicine of the National University of Singapore, and the Program Director of the Infectious Diseases Fellowship Training Program at the National University Health System, Singapore. She has been actively involved in Singapore’s transition to ACGME-International accreditation since 2009. She is active in teaching learners across the medical education continuum, especially residents-as-teachers.
