# Supplementary material for: Practical tips for bedside teaching of infectious diseases practice to junior doctors
Source: MedEdPublish (2016). 2017 Oct 11;6:180. [Version 1] doi: 10.15694/mep.2017.000180 (PMC10885302; doi:10.15694/mep.2017.000180)
Supplement: Peer review history

# Reviewer report: Reviewer response for version 1

**DOI:** 10.21956/mep.19360.r28321

**Published:** 2017-10-17

**Authors:** Ralph Pinnock

**Affiliations:** 1 University of Orago, Dunedin, New Zealand

This review has been migrated. The reviewer awarded 3 stars out of 5 Like the other reviewers I found this a helpful paper based on sound educational principles. Did student feedback provide any helpfil insightsRalph Pinnock

Reviewer Expertise:

NA

No decision status is available

# Reviewer report: Reviewer response for version 1

**DOI:** 10.21956/mep.19360.r28318

**Published:** 2017-10-12

**Authors:** Nandalal Gunaratne

**Affiliations:** 1 Faculty of Medicine, Wayamba University of Sri Lanka

This review has been migrated. The reviewer awarded 3 stars out of 5 This is a good approach to teaching. I have followed a similar approach and now I have added some of your recommendations for future teaching. The most difficult is the fourth step. Step 4. What are the suspect culprit pathogens? (Linking microbiology at bedside)Students are a bit overwhelmed by this as they have learnt many pathogens, subtypes and serotypes. They also argue that in the clinical setting it is unecessary as it is a job for the lab to identify the pathogen and say which antibiotic is appropriate! Their job is to collect and send the right specimen in the correct way!Regards.

Reviewer Expertise:

NA

No decision status is available

# Reviewer report: Reviewer response for version 1

**DOI:** 10.21956/mep.19360.r28319

**Published:** 2017-10-11

**Authors:** Lucy Frost

**Affiliations:** 1 Oxford University

This review has been migrated. The reviewer awarded 4 stars out of 5 This article proposes a model for bedside teaching in infectious diseases that builds on previous educational theories. It concisely sets out the model, providing enough information and justification for it to be followed, but without over-complicating it. This makes it easy to access and use. The steps suggested follow strong medical educational theories, and would be adaptable to other contexts. There are a few grammatical errors, which I am happy to expand on if you are interested! Overall, I find this paper a useful contribution and one that is readily applicable to many readers involved in bedside teaching in a range of specialities.

Reviewer Expertise:

NA

No decision status is available

# Reviewer report: Reviewer response for version 1

**DOI:** 10.21956/mep.19360.r28320

**Published:** 2017-10-11

**Authors:** John Dent

**Affiliations:** 1 AMEE

This review has been migrated. The reviewer awarded 4 stars out of 5 Thank you for this practical paper describing a useful approach to structuring a bedside teaching session in your discipline.I liked your three-part rationale and the nine-step approach to organising the teaching session. I think that a technique which helps the teacher to teach will also help the learner to learn. Too often bedside teaching is approached without thoughtful preparation or formal structure. I think your paper highlights a good approach which others may wish to try in their own clinical discipline.

Reviewer Expertise:

NA

No decision status is available
